# MSAL-Net: improve accurate segmentation of nuclei in histopathology images by multiscale attention learning network

**DOI:** 10.1186/s12911-022-01826-5

**Published:** 2022-04-04

**Authors:** Haider Ali, Imran ul Haq, Lei Cui, Jun Feng

**Affiliations:** School of Information Science and Technology, Northwest University, Xian, China

**Keywords:** Deep learning, Nuclei segmentation, Multiscale attention learning, Convolutional neural network

## Abstract

**Background:**

The digital pathology images obtain the essential information about the patient’s disease, and the automated nuclei segmentation results can help doctors make better decisions about diagnosing the disease. With the speedy advancement of convolutional neural networks in image processing, deep learning has been shown to play a significant role in the various analysis of medical images, such as nuclei segmentation, mitosis detection and segmentation etc. Recently, several U-net based methods have been developed to solve the automated nuclei segmentation problems. However, these methods fail to deal with the weak features representation from the initial layers and introduce the noise into the decoder path. In this paper, we propose a multiscale attention learning network (MSAL-Net), where the dense dilated convolutions block captures more comprehensive nuclei context information, and a newly modified decoder part is introduced, which integrates with efficient channel attention and boundary refinement modules to effectively learn spatial information for better prediction and further refine the nuclei cell of boundaries.

**Results:**

Both qualitative and quantitative results are obtained on the publicly available MoNuseg dataset. Extensive experiment results verify that our proposed method significantly outperforms state-of-the-art methods as well as the vanilla Unet method in the segmentation task. Furthermore, we visually demonstrate the effect of our modified decoder part.

**Conclusion:**

The MSAL-Net shows superiority with a novel decoder to segment the touching and blurred background nuclei cells obtained from histopathology images with better performance for accurate decoding.

## Introduction

Cancer grading has an essential importance in clinics because it allows doctors to treat patients more effectively and analyze the effectiveness of treatments. The microscopic images are broadly utilized to diagnose tumors in clinical medicine, consists of vital information about encompassing the tissue structure and tumors. The particular structures of tissue such as glands, collagen and nuclei are stained to highlight. The hematoxylin and eosin (H&E) staining is the most common method for separating nuclei and cytoplasm in slices. The characteristics of nuclei are used by clinicians to grade tumor, and in the slices, morphology and nucleus polymorphism are observed.

**Fig. 1:**
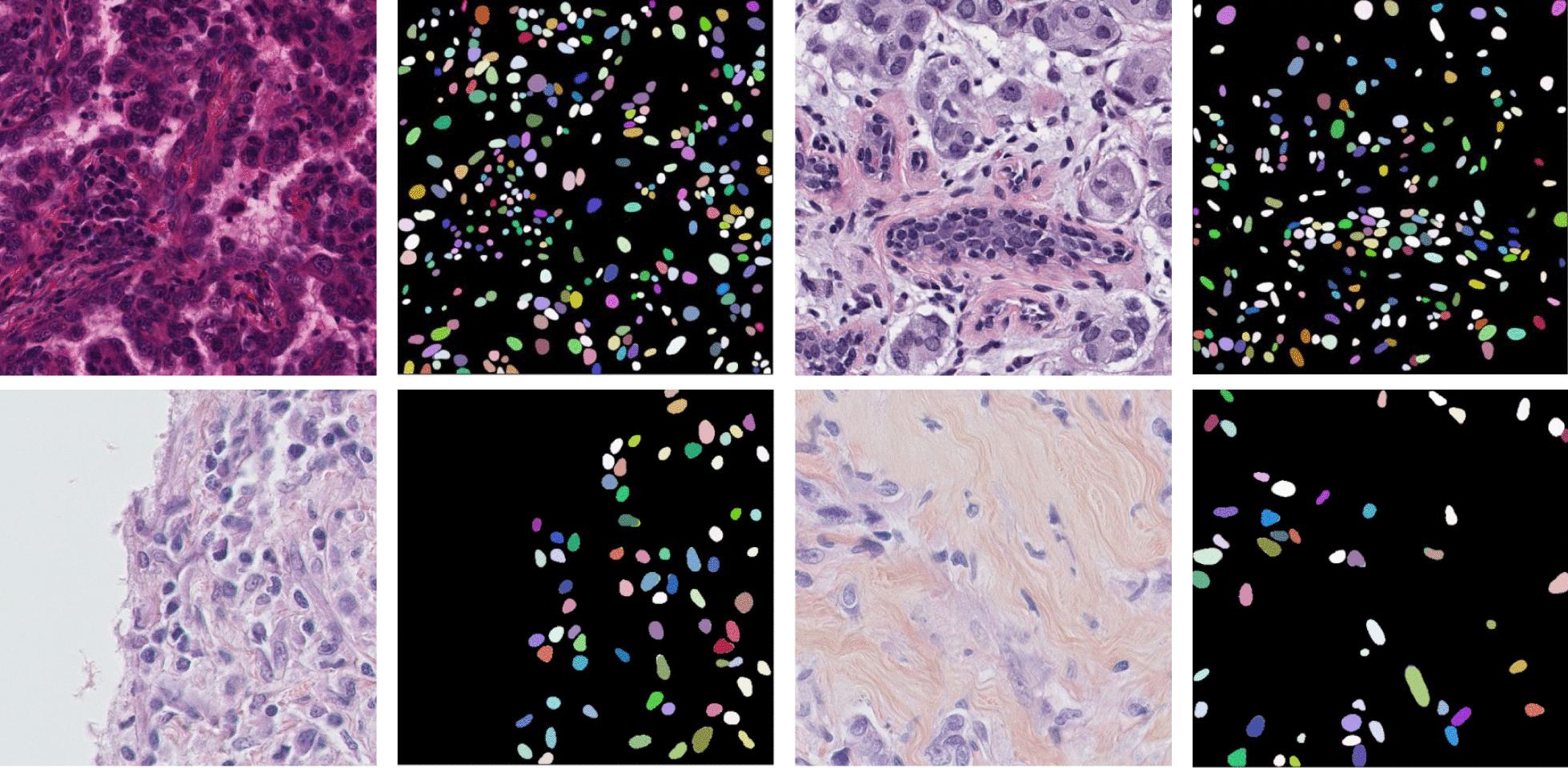
Show some examples of original pathology images in two different datasets, the first row represents the MoNuSeg dataset images [12], and the second row represents the TNBC dataset images [13]

Digital pathology has gained significant research focus in the medical field due to the constant advancement of software and hardware proficiencies. Whole slide images (WSIs) can be obtained using high-intelligence and automation scanning devices to create an advanced database of digital pathology. Computerized strategies have proven effective in explaining a wide range of pathology issues for analyzing histopathological images [1, 2]. Nuclear characteristics are crucial parameters for cancer grading during diagnoses, such as form, texture, and spatial arrangement. For a computer-aided diagnosis framework to quantify cellular morphology in cancer diagnosis, accurate nuclei segmentation is a key and fundamental step [3]. Manual segmentation of the nuclei in sliced tissue is used in traditional cancer diagnosis, although this method is tedious and prone to false positives given a large number of nuclei. Thus, implementing an automated nuclei segmentation method in digital pathology image processing remains problematic due to the nucleus occlusion and overlapping, shape variations, image artefacts, and blurred background [4]. The classical nuclei strategies based on thresholding [5], region splitting and merging [6], clustering [7], watershed segmentation [8], graph-based segmentation [9], pixel classification [10], level set [11] and so on. Due to the relevance of nucleus information in medicine, many researchers have proposed different approaches for pathology images segmentation. These methods are unsuccessful when applied to pathology images, and Fig. 1 shows some examples of nuclei pathology images and corresponding segmentation masks.

Recently, deep learning algorithms such as convolutional neural networks (CNN) [14] and autoencoders [15] have gained a more scientific research value in computer vision, especially in recognition and image segmentation. Compared to traditional image segmentation methods, these algorithms have been widely applied in medical image analysis. CNN techniques demonstrate remarkable outcomes for digital pathology image analysis, including tissue classification [16], mitotic detection [17, 18] and nuclei segmentation [19–21]. The fundamental difficulties with CNN models are that they are based on a fully convolutional network (FCN) [22] that reduces the resolution of the feature maps by using the downsampling operations to obtain rich semantic information. FCN raises the resolution of the feature maps using upsampling operations, but it also results in the loss of certain target object features when the final output resolution is set to the same as the original image. In order to overcome this limitation, Ronneberger et al. [23] proposed an Unet method based on FCN, which used skip connections to combine the spatial information from the encoder path with the decoder path to retain good spatial information. However, this process brought the poor feature representation from the initial layers and introduced the noise into the decoder. Kong et al. [24] proposed a two-stage stacked Unets, where the first stage aimed to segment nuclei regions, and the second stage was considered to segment regions of overlapping nuclei. The stacked Unets were successful in segmenting nuclei. However, training was difficult due to the high experimental hardware requirements. Inspired by [25], He et al. [26] proposed a Hybrid Nested-Unet, which employed UNet’s nested multiple convolution blocks to bridge the possible semantic gap between the encoder-decoder levels in classic Unet. However, it was better to introduce a new pipeline instead of increasing the model complexity. Recently, some methods were introduced based on attention mechanisms [27–29], which allowed the network to focus on the meaningful features between the encoder and decoder. However, these methods were confirmed to be crucial for the accurate decoder in the nuclei segmentation task and Later made segmentation of touching and overlapping nuclei problematic. Notably, during H&E staining and scanning, the boundaries of nuclei in pathology datasets may become blurred.

**Fig. 2:**
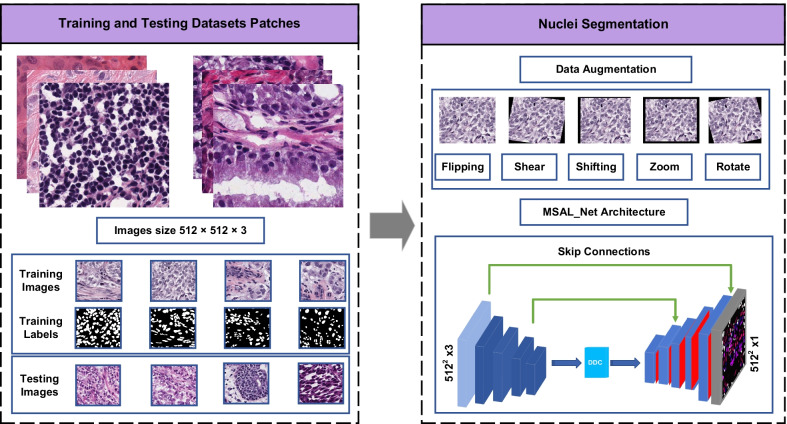
Displays the overview of our proposed method for nuclei segmentation on pathology images

In recent years, transfer learning has been a beneficial method and widely applied in medical image tasks when the training samples are in shortage. However, deep learning algorithms prone to overfitting on the small training samples and the time-consuming image labelling task solve this problem [30–34]. In contrast, our proposed method effectively alleviates this obstacle and increases network performance by decomposing training images into small patches as well as using the data augmentation technique. The overview of our proposed multiscale attention learning network (MSAL-Net) for accurate nuclei segmentation is presented in Fig. 2. An encoder block in the network extracts the semantic and spatial information from the input of histopathology images. The dense dilated convolutions (DDC) are then combined in order to generate more high-level feature maps. Finally, the decoder path learns spatial information and recovers the touching and overlapping of complicated nuclei object boundaries without blurred background, which can be easily overlooked or identified mistakenly using some standard image segmentation methods. Compared with other methods, MSAL-Net has achieved better segmentation results and performance in nuclei segmentation tasks. The extensive experimental results demonstrate the effectiveness of our proposed method.

The contribution of this research study are: (1) We employ the residual blocks in the encoder to improve the feature extraction capability of the network. (2) In order to generate more high-level features maps and reduce the semantic gap between the encoder and decoder, we add a dense dilated convolution (DDC) block in the middle of the network. (3) We present an idea of a novel decoder path that combines with the ECA and BR modules to learn the essential feature map, refine the information of nuclei boundaries, and significantly enhance the network performance for accurate decoding. (4) we combine the encoder-decoder architecture with the DDC and ECA + BR modules for nuclei segmentation in histopathology images is present. (5) The experiments have been carried out on the public nuclei histopathology dataset. Additionally, some selected advanced semantic segmentation approaches compared with the proposed model and the outcomes of all experiments are thoroughly examined. The experimental results reveal that our method learns robust features and produces an outstanding effect in the segmentation. Besides, our proposed model also compared with the current state-of-the-art approaches.

## Methodology

### Proposed method architecture

#### Overview

**Fig. 3:**
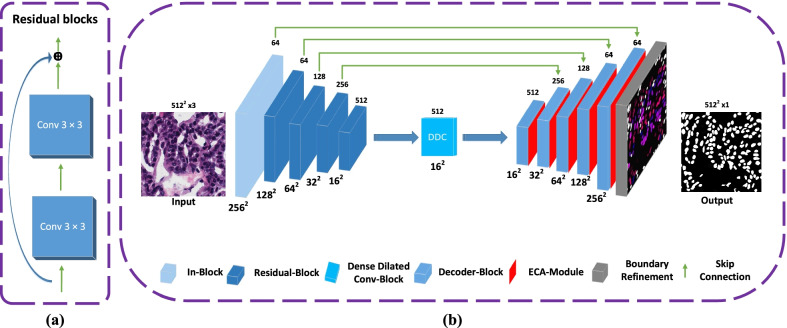
**a** Demonstrates the residual blocks, where each block consists of $3 \times 3$ convolution and **b** shows the network architecture, where the downsampling layers (In-Block, Residual-Blocks) receive the input of RGB image, then DDC is applied in the middle part, and upsampling layers (Decoder-Block) deliver the final output. The green arrows represent the skip connections between the contraction to expansion path. Furthermore, each convolutional block uses a conv, batchnorm, and relu layer, whereas each deconvolutional block uses a deconv, batchnorm and relu layer

Motivated by the ResNet [35], Inception Net [36], Dilated Convolutions [37], Efficient Channel Attention (ECA) [38] and U-net [23], we introduced a novel method for accurate nuclei segmentation called MSAL-Net, as shown in Fig. 3b. Initially, the encoder structure extracted the spatial information from the input of histopathology images, and the DDC block was used to extract high-level feature maps in the middle part of the network. The decoder structure learned spatial information for better prediction and strengthened the nuclei boundaries.

#### Encoder structure

In our proposed method, the structure of the encoder was modified from the Unet design and replaced it with the In-Block, 4 Residual-Blocks of Resnet 34 [35] pretrained from ImageNet, while the original Unet encoder of each block consisted of convolution layers and max-pooling layers. The first input of histopathology image passed through the initial encoder In-Block of the network, which performed convolutional block and max-pool layers at input features such as 3 $\times$ 512 $\times$ 512 with a kernel size of 7 $\times$ 7 and stride of 2. The max-pool layer reduced the feature map to half its original input size. Subsequently, there was 4 Residual-Blocks without the average pooling and the fully connected layers, which was lighter, more efficient in computation and accuracy. Each Residual-Block doubled the feature channel and scaled down the image by a variable of two. These Residual-Blocks allowed the network to better aggregate information between encoder and decoder layers. Besides, Residual-Blocks used a shortcut to avoid gradient vanishing and enhanced the network convergence, as shown in Fig. 3a.

#### Dense dilated convolutions

**Fig. 4:**
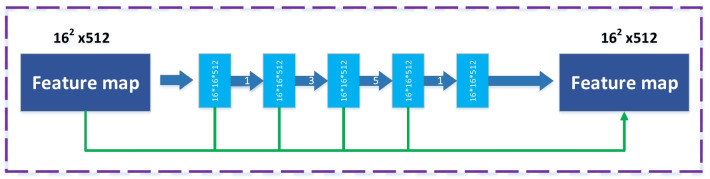
Illustrates the dense convolutions block, which is based on cascade mode [37]

In semantic segmentation tasks, the pooling layers caused the loss of semantic information in images [39]. Inspired by the dilated convolutions and Inception series structures, we use the DDC block in the middle part of our network to encode the high-level semantic feature maps, as depicted in Fig. 4. The encoder structure contains five downsampling layers, and if an image of a size 512 $\times$ 512 passes through it, the output feature map size will be 16 $\times$ 16 pixels. If the stacked DDC are from 1 to 1, 3, 5, respectively, then the receptive field of every layer will be 3, 7, 9, 19. Each dilated convolutional layer receives one 1 $\times$ 1 convolution for rectified linear activation. Finally, we incorporate into the original features other elements, such as the ResNet shortcut mechanism. The DDC enhances the receptive fields in the network and does not reduce the resolution of the feature maps.

#### Decoder structure

**Fig. 5:**
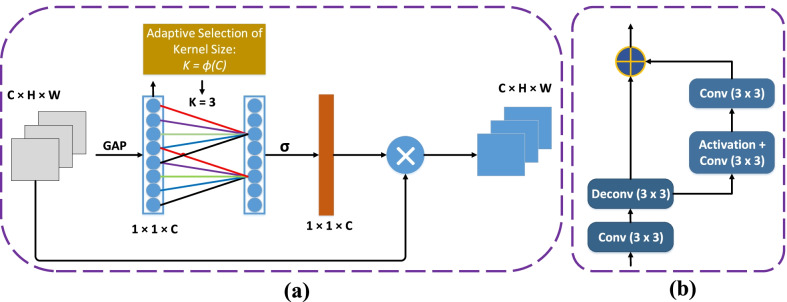
**a** Shows the diagram of the ECA module, where the GAP denotes the global average pooling, k represents the kernel size, C, H, and W represent the channel, height and width, denotes the sigmoid [38], and **b** displays the boundary refinement module, where 3 $\times$ 3 represents the convolutional filter size

The decoder was used to retrieve the combined spatial information from the encoder and dense dilated convolutions block. However, skip connection brought the poor feature representation from the initial layers and introduced the noise into the decoder. Similar to [40], each block of the decoder reduced the number of feature channels by half and increased the image size by a factor of two, as illustrated in Fig. 3b. After each decoder block, we employed a parallel efficient channel attention (ECA) module of the same dimension as shown in Fig. 5a. ECA used 1D convolution to construct a local cross channel interaction method that required dimensionality reduction and could be done efficiently. ECA suppressed less significant feature maps and enhanced overall performance via boosting up essential features. The segmentation results were better predicted and refined using the boundary refinement (BR) module [41] after the decoder block. The BR module built residual structure by applying three convolution filter sizes from extracted features, as shown in Fig. 5. The sigmoid function was used to predict the final segmentation outcome.

### Results

#### Dataset

The MoNuseg dataset consists of 44 pathological tissue slices and is stained with H&E captured at 40x magnification. Paches sampled from whole slide images with a fixed size of 1000 $\times$ 1000 pixels, these sample images have taken out from multiple organs and collected from 18 different hospitals. There are 30 images in the training set, and 14 images are in the testing set. The training set manually labeled nuclei are 21,623, and the testing set manually labelled nuclei are 21,000. Moreover, the images are marked by experienced pathologists with the associated ground truth [12]. Subsequently, patches are resized to 512 $\times$ 512 pixels from 4 corners of each image [42] and fed into the MSAL-Net. There are 176 patches in total, and 120 patches are used for training, and 56 patches are used for testing.

#### Implementation details

The proposed MSAL-Net was built by PyTorch [43] deep learning library and tested it on the GPU GTX 1080 Ti with 16 GB of memory. The input images are resized by 512 $\times$ 512 and then fed into the network. During the training and testing phases, we employed batch size 2 and trained the network with 300 epochs. The learning rate was set to 0.001 with the Adam optimizer, and the network employed a pre-trained ResNet-34 structure. A decay rate of 0.00003 was used to mitigate cross-entropy loss in nucleus segmentation tasks. A variety of data augmentation techniques were used on the training set, including random horizontal and vertical flipping, random rotation from -15 to 15, random shifting in x and y directions, random shearing, and random zooming from 0.0 to 0.2, as illustrated in Fig. 2.

#### Performance metrics

The four metrics used to evaluate the effectiveness of our proposed method in the nuclei segmentation tasks such as Dice coefficient (DC), Intersection over Union (IoU) Precision (PR), and Recall (RC). These matrices formulas can be computed as:

First, let say $A=TP$ and $B = TP + FP + FN$

Now, we have

1 $$\begin{aligned} {\text {Dice}}(A, B)&= \frac{2|A \cap B| }{|A|+|B|} \end{aligned}$$

2 $$\begin{aligned} {\text {IoU}}(A, B)&= \frac{|A \cap B| }{|A\cup B||} \end{aligned}$$

3 $$\begin{aligned} {\text{PR}}&= \frac{{\mathrm{TP}}}{{\mathrm{TP}}+{\mathrm{FP}}} \end{aligned}$$

4 $$\begin{aligned} {\text{RC}}&= \frac{{\mathrm{TP}}}{{\mathrm {TP}}+{\mathrm {FN}}} \end{aligned}$$

**Fig. 6:**
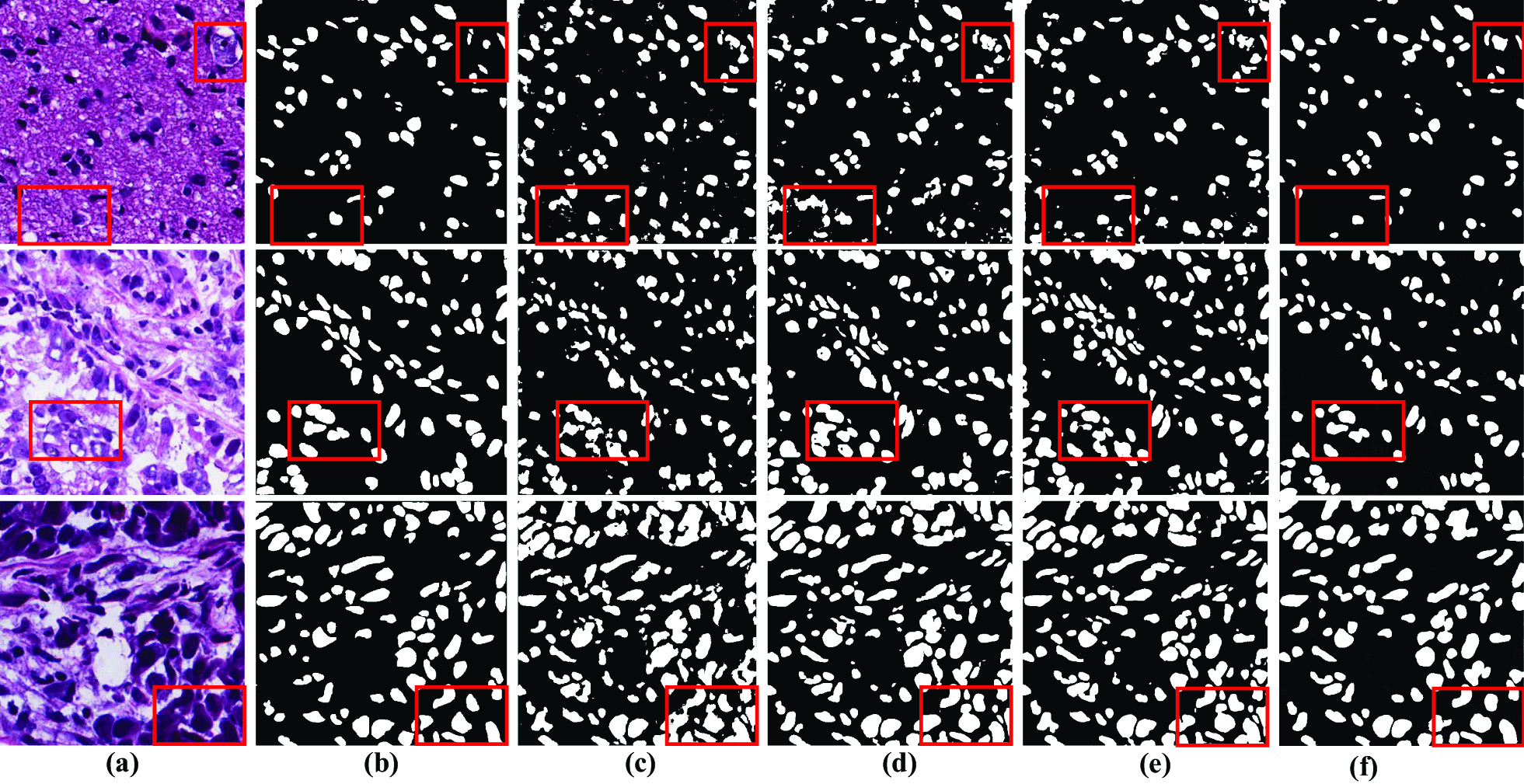
Shows the comparison with other methods through red boxes **a** input image, **b** ground truth, **c** U-net results, **d** Attention U-net results, **e** CE-Net results, and **f** proposed MSAL-Net results

The DC and IoU metrices values will be between 0 and 1. The $\cup ,\cap$ symbols signify the mathematical operations union and intersection. The TP, FP, and FN are used to calculate the pixel level difference between the expected image and the ground truth target. Moreover, TP, FP and FN represent the number of correctly segmented pixels, the number of pixels that appear in the segmentation results, and the number of pixels that appear in the ground truth, respectively (Fig. 6).

#### Ablation study

**Fig. 7:**
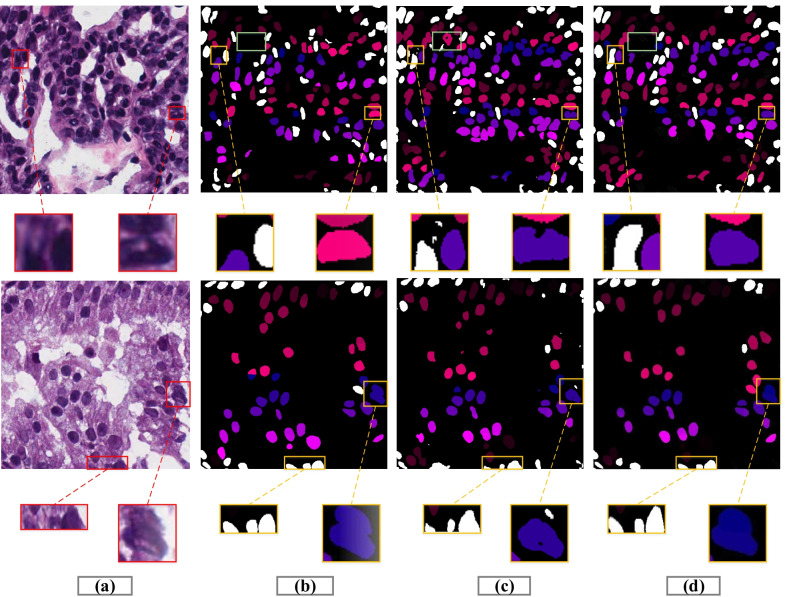
Represents some segmentation examples of decoder, **a** input image, **b** ground truth, **c** result of MSAL without ECA + BR modules, **d** and the result of MSAL with ECA + BR modules

**Fig. 8:**
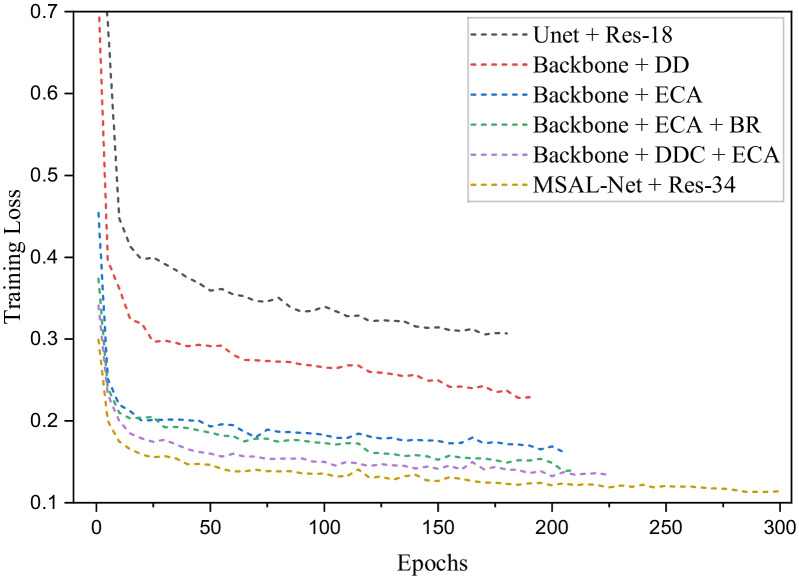
Training loss of different models

This section explained the ablation study to demonstrate the efficacy of each component in the MSAL-net on the test set, as indicated in Table 1. The different trained components of our model results, such as, Backbone + DDC, Backbone + ECA, Backbone + Res-34 + DDC, Backbone + ECA + BR, Backbone + DDC+ ECA, and Backbone + Res-18 + DDC + ECA + BR were listed in the first row. The k denoted the size of the convolutional filters used in the ECA and BR modules to extract data, while the “–” symbol signified missing values. The evaluation metrics results of each component were represented in the next four parts of the table, and the last part reflected the computational training time in seconds. Unet is one of the essential frameworks in medical images segmentation problems, and it was the cornerstone for our proposed approach (Fig. 7). The encoder element of the network was replaced by residual operations (ResNet 34 block), which allowed it to train deeper and increase learning capabilities such as backbone + ResNet outperformed the baseline with a DC of 0.79. The dense dilated convolutions block was stacked at different rates to enlarge the receptive field of view, reduce the loss of image information, extract high-level features and improve performance 0.810 DC (Backbone + DDC). We found that the both ECA module with backbone and ECA + BR with backbone improved performance and segmentation significantly. Besides, when compared to other Res-18, Res-50, and Res-101 backbones, MSAL-Net with Res-34 achieved efficient outcomes at DC 0.839 and IoU 0.706 during training. The proposed model enhanced DC performance by 1.5 percent and IoU performance by 1 percent with the Res-50 backbone. Thus, MSAL-Net with Res-101 reduced performance by 0.09 percent in DC and 1 percent in IoU compared to Res-50. However, these two backbones increased the network’s training time and made it slow. Besides, we recorded the loss changes of different combinations for the proposed model during the training process, as displayed in Fig. 8. We observed from Fig. 8 that the training losses of these combinations Backbone + ECA, Backbone + ECA + BR, Backbone + DDC+ ECA, and DDC + ECA + BR + Res- 34 (MSAL-Net) remained stable compared to others, which proved the proposed model good convergence, generalization and stability.

**Table 1:** Represents the model comparison of each component on the test set, and k denotes the convolutional filters are used to extract features

Method	MoNuseg test set					
	K	DC	IOU	PR	RC	Training Time
Unet [23] + Res-18 [35]	–	0.781	0.642	0.774	0.792	211.9
Backbone + Res-34 [35]	–	0.793	0.652	0.785	0.801	–
Backbone + DDC	–	0.810	0.682	0.783	0.845	–
Backbone + Res-34 + DDC	–	0.817	0.692	0.790	0.837	–
Backbone + ECA	3	0.809	0.681	0.778	0.829	–
Backbone + ECA + BR	3	0.815	0.688	0.805	0.827	–
Backbone + DDC + ECA	3	0.820	0.696	0.809	0.848	–
Backbone + Res-18 + DDC + ECA + BR	3	0.829	0.699	0.813	0.846	181.3
MSAL-Net + Res-34	3	0.839	0.706	0.821	0.853	194.5
MSAL-Net + Res-50	3	0.847	0.713	0.816	0.881	317.7
MSAL-Net + Rest-101	3	0.830	0.695	0.801	0.862	429.1

#### The effect of ECA and BR with decoder

The proposed method demonstrated both qualitative and quantitative results to evaluate the effect of the ECA and BR modules with the decoder part, as illustrated in Fig. 7 and Table 1. In nuclei segmentation tasks, the histopathological images contained complicated cases with higher variability in forms and nuclei boundaries in pathology datasets may become blurred. Thus, it was hard for the network with (backbone + DDC) to segment the nuclei without noise and refine the boundaries of nuclei cells, as shown in Fig. 7c. Next, we observed that the ECA module with the network reduced the noise and gained performance by suppressing less significant features, but some nuclei cell shapes remained missing. Ultimately, the residual-based BR and ECA modules worked efficiently to refine the exact boundaries of nuclei cells and increased the localization performance, as shown in Fig. 7d. Additionally, our network showed superior performance for accurate decoding in nuclei segmentation tasks.

#### Computation analysis

We evaluated the computational cost of segmentation on a machine with an NVIDIA GeForce GTX 1080 Ti GPU. The nuclei histopathology images with a size of 512 $\times$ 512 were used in all the experiments and the computational times reported in Table 1. Our suggested MSAL-Net with Res-34 produced the best results with the highest efficacy compared to alternative backbones. We also noted that the MSAL-Net with Res-50 improved the performance but took an amount of training time. Furthermore, as shown in Table 2, our proposed MSAL-Net was computationally efficient than other advanced segmentation methods.

**Table 2:** Compared results with state-of-the-art approaches on the test set

Method	DC	IoU	PR	RC	Training Time
LinkNet [40]	0.767	0.625	0.726	0.809	178.3
Unet [23]	0.773	0.548	0.758	0.789	211.9
CE-Net [44]	0.818	0.693	0.809	0.829	271.1
Attention-Unet [27]	0.810	0.678	0.779	0.848	480.6
ASSPU-Net [45]	0.830	0.780	–	–	1133.7
MDC-Net [46]	0.802	–	–	–	–
Bio-Net [42]	0.826	0.704	–	–	–
CNN3 [13]	0.804	0.498	–	–	–
MSAL-Net	0.839	0.706	0.821	0.853	194.5

The “–” symbol denotes the missing values

## Discussion

We obtained results using other methods compared qualitatively and quantitatively. Figure 6 illustrates the visually compared results with three state-of-the-art deep learning methods via three examples of histopathological images segmented and the red boxes indicated the differences. The Unet, Attention-Unet and CE-Net methods yielded poor results by examining the figures, such as introduced noise and missed or wrongly identified nuclei. This was because these methods combined spatial information from the encoder path with the decoder path to complementary spatial information via ski connections. However, this procedure introduced noise into the decoder by bringing the poor feature representation from the earlier layers into the decoder. In the multi-organ histopathological images, the nuclei contained complicated cases with higher heterogeneity in forms and textures. Therefore, these methods could not correctly segment the nuclei and failed to detect the nuclei location and clear boundaries due to the decoder’s weak feature representation. Compared to Unet, Attention-Unet, and CE-Net, our MSAL-Net approach effectively and accurately segmented nuclei, refined the borders of complicated histopathological images at the nuclei level, and produced good segmentation results.

Apart from the visual comparison, the performance of our proposed method compared to the first four selected state-of-the-art segmentation methods displayed in Table 2, such as LinkNet, Unet, Attention-Unet, and CE-Net. The four widely performance metrics DC, IoU, PR and RC were used to evaluate the performance of the selected methods along with the proposed method. By observing the MSAL-Net ablation experimental evaluation in Table 1, the proposed method outperformed selected methods in all DC 0.839, IoU 0.706, PR 0.821, and RC 0.853 metrics. In addition, we discovered that the lightweight LinkNet approach took less training time (178.3) than Unet but performed poorly on the MoNuseg test set. Attention-Unet outperformed Unet and LinkNet in terms of DC 0.810, IoU 0.678, PR 0.779, and RC 0.848, but it performed worse in IoU 0.678 and took longer to train (480.6) than CE-Net. Our proposed technique outperformed CE-Net, Attention-Unet, and Unet in terms of DC 0.839 and IoU 0.706. In contrast, we compared our method to other recent advanced segmentation approaches, which had shown efficient performance in nuclei segmentation tasks, such as Bio-Net achieved 0.826 DC, MDC-Net reached 0.800 DC, CNN3 reached 0.804 DC and ASSPU-Net achieved 0.830 DC. They are computationally ineffective in nuclei segmentation due to their complex architecture and high training time requirements. Extensive experiments revealed that our proposed MSAL-Net outperformed all comparison models, reported the best results in Table 2 and showed greater efficacy in nuclei segmentation, as shown in Fig. 6.

Our research study proposed a model to tackle the accurate segmentation of nuclei in histopathology images. Our model introduced an idea of a novel decoder that was integrated with the attention and refinement modules. We tested our model through four performance metrics on the MoNuseg test set and compared it with the current deep learning mainstream approaches. The extensive experiment results visually and quantitatively demonstrated the feasibility and superiority of the proposed MSAL-Net in nuclei segmentation. We also carried out an ablation test on each component of our proposed technique and analyzed the effectiveness of the ECA and BR modules with the decoder. The above study claimed that the proposed method could effectively segment the nuclei, while still there was potential for improvement. Besides, we believe that the proposed MSAL-Net can be helpful for other segmentation problems.

## Conclusion

This study aimed to introduce a method to improve the accurate segmentation of nuclei in histopathology images. In this paper, we presented a method called MSAL-Net for the nuclei segmentation task, which employed residual blocks in the encoder structure to improve the feature extraction capability and used the DDC block to capture broader nuclei context information. The ECA and BR modules were integrated with decoder structure to learn spatial information for better prediction and refine the information of nuclei boundaries. Extensive experiments demonstrated that the proposed MSAL-Net outperformed other state-of-the-art methods in visual perception and quantitative metrics using the publicly available MoNuseg dataset. Furthermore, though the proposed MSAL-Net was designed to solve the nuclei segmentation problem, we believe it may also be effective for other segmentation challenges as well, which we want to investigate further in the future study.

## Data Availability

This study used publicly available dataset MoNuSeg 2018 MICCIA challenge [12] can be found on this website: https://monuseg.grand-challenge.org. The training set and test set can be accessed by emailing the first author that support the conclusions of this article. Project homepage: https://github.com/haideralimughal/MSAL-Net.

## Abbreviations

CNN: Convolutional neural networks
MASAL-Net: Multiscale attention learning network
ECA: Efficient channel attention
BR: Boundary refinement
HE: Hematoxylin and eosin
WSI: Whole slide images
DDC: Dense dilated convolutions
DC: Dice coefficient
IoU: Intersection over Union
PR: Precision
RC: Recall
TP: True positive
FP: False negative
FN: False negative

## References

[1] Hou L, Nguyen V, Kanevsky AB, Samaras D, Kurc TM, Zhao T, Gupta RR, Gao Y, Chen W, Foran D (2019). Sparse autoencoder for unsupervised nucleus detection and representation in histopathology images. Pattern Recogn.

[2] Wan T, Zhang W, Zhu M, Chen J, Achim A, Qin Z (2017). Automated mitosis detection in histopathology based on non-Gaussian modeling of complex wavelet coefficients. Neurocomputing.

[3] Nir G, Hor S, Karimi D, Fazli L, Skinnider BF, Tavassoli P, Turbin D, Villamil CF, Wang G, Wilson RS (2018). Automatic grading of prostate cancer in digitized histopathology images: learning from multiple experts. Med Image Anal.

[4] Pan X, Li L, Yang H, Liu Z, Yang J, Zhao L, Fan Y (2017). Accurate segmentation of nuclei in pathological images via sparse reconstruction and deep convolutional networks. Neurocomputing.

[5] Xue J-H, Titterington DM. $$t$$-tests, $$f$$-tests and otsu’s methods for image thresholding. IEEE Trans Image Process. 2011;20(8):2392–6. 10.1109/TIP.2011.2114358 21324779

[6] Basavanhally AN, Ganesan S, Agner S, Monaco JP, Feldman MD, Tomaszewski JE, Bhanot G, Madabhushi A (2009). Computerized image-based detection and grading of lymphocytic infiltration in her2+ breast cancer histopathology. IEEE Trans Biomed Eng.

[7] Clark MC, Hall LO, Goldgof DB, Clarke LP, Velthuizen RP, Silbiger MS (1994). MRI segmentation using fuzzy clustering techniques. IEEE Eng Med Biol Mag.

[8] Veta M, Van Diest PJ, Kornegoor R, Huisman A, Viergever MA, Pluim JP (2013). Automatic nuclei segmentation in h&e stained breast cancer histopathology images. PLoS ONE.

[9] Kost H, Homeyer A, Molin J, Lundström C, Hahn HK. Training nuclei detection algorithms with simple annotations. J Pathol Inform, 2017;8. 10.4103/jpi.jpi_3_17 PMC5450511 28584683

[10] Liu T, Guo Q, Lian C, Ren X, Liang S, Yu J, Niu L, Sun W, Shen D (2019). Automated detection and classification of thyroid nodules in ultrasound images using clinical-knowledge-guided convolutional neural networks. Med Image Anal.

[11] Guo P, Evans A, Bhattacharya P. Segmentation of nuclei in digital pathology images. In: 2016 IEEE 15th international conference on cognitive informatics & cognitive computing (ICCI* CC), 2016;547–550. IEEE.

[12] Kumar N, Verma R, Sharma S, Bhargava S, Vahadane A, Sethi A (2017). A dataset and a technique for generalized nuclear segmentation for computational pathology. IEEE Trans Med Imaging.

[13] Naylor P, Laé M, Reyal F, Walter T (2018). Segmentation of nuclei in histopathology images by deep regression of the distance map. IEEE Trans Med Imaging.

[14] Gu J, Wang Z, Kuen J, Ma L, Shahroudy A, Shuai B, Liu T, Wang X, Wang G, Cai J (2018). Recent advances in convolutional neural networks. Pattern Recogn.

[15] Bank D, Koenigstein N, Giryes R. Autoencoders. arXiv preprint arXiv:2003.05991; 2020.

[16] Wang M, Lian C, Yao D, Zhang D, Liu M, Shen D (2019). Spatial-temporal dependency modeling and network hub detection for functional MRI analysis via convolutional-recurrent network. IEEE Trans Biomed Eng.

[17] Deepak S, Ameer P (2019). Brain tumor classification using deep CNN features via transfer learning. Comput Biol Med.

[18] Bria A, Marrocco C, Tortorella F (2020). Addressing class imbalance in deep learning for small lesion detection on medical images. Comput Biol Med.

[19] Liu X, Fu T, Pan Z, Liu D, Hu W, Liu J, Zhang K (2018). Automated layer segmentation of retinal optical coherence tomography images using a deep feature enhanced structured random forests classifier. IEEE J Biomed Health Inform.

[20] Lal S, Das D, Alabhya K, Kanfade A, Kumar A, Kini J (2021). Nucleisegnet: robust deep learning architecture for the nuclei segmentation of liver cancer histopathology images. Comput Biol Med.

[21] Xie L, Qi J, Pan L, Wali S (2020). Integrating deep convolutional neural networks with marker-controlled watershed for overlapping nuclei segmentation in histopathology images. Neurocomputing.

[22] Long J, Shelhamer E, Darrell T. Fully convolutional networks for semantic segmentation. In: Proceedings of the IEEE conference on computer vision and pattern recognition, 2015;3431–3440. 10.1109/TPAMI.2016.2572683 27244717

[23] Ronneberger O, Fischer P, Brox T. U-net: Convolutional networks for biomedical image segmentation. In: International conference on medical image computing and computer-assisted intervention, 2015;234–241. Springer.

[24] Kong Y, Genchev GZ, Wang X, Zhao H, Lu H (2020). Nuclear segmentation in histopathological images using two-stage stacked u-nets with attention mechanism. Front Bioeng Biotechnol.

[25] Zhou Z, Siddiquee MMR, Tajbakhsh N, Liang J. Unet++: A nested u-net architecture for medical image segmentation. In: Deep learning in medical image analysis and multimodal learning for clinical decision support, pp. 3–11. Springer, 2018. 10.1007/978-3-030-00889-5_1 PMC7329239 32613207

[26] He H, Zhang C, Chen J, Geng R, Chen L, Liang Y, Lu Y, Wu J, Xu Y (2021). A hybrid-attention nested unet for nuclear segmentation in histopathological images. Front Mol Biosci.

[27] Oktay O, Schlemper J, Folgoc LL, Lee M, Heinrich M, Misawa K, Mori K, McDonagh S, Hammerla NY, Kainz B, et al. Attention u-net: learning where to look for the pancreas. arXiv preprint arXiv:1804.03999; 2018.

[28] Guo C, Szemenyei M, Yi Y, Wang W, Chen B, Fan C. Sa-unet: Spatial attention u-net for retinal vessel segmentation. In: 2020 25th international conference on pattern recognition (ICPR), 2021;1236–1242. IEEE.

[29] Lian S, Luo Z, Zhong Z, Lin X, Su S, Li S (2018). Attention guided u-net for accurate iris segmentation. J Vis Commun Image Represent.

[30] Oquab M, Bottou L, Laptev I, Sivic J. Learning and transferring mid-level image representations using convolutional neural networks. In: Proceedings of the IEEE conference on computer vision and pattern recognition, 2014;1717–1724.

[31] Wollmann T, Ivanova J, Gunkel M, Chung I, Erfle H, Rippe K, Rohr K (2018). Multi-channel deep transfer learning for nuclei segmentation in glioblastoma cell tissue images. Bildverarbeitung für die Medizin.

[32] Wahab N, Khan A, Lee YS (2019). Transfer learning based deep CNN for segmentation and detection of mitoses in breast cancer histopathological images. Microscopy.

[33] Bayramoglu N, Heikkilä J. Transfer learning for cell nuclei classification in histopathology images. In: European conference on computer vision, 2016;532–539. Springer.

[34] Chang J, Yu J, Han T, Chang H-j, Park E. A method for classifying medical images using transfer learning: a pilot study on histopathology of breast cancer. In: 2017 IEEE 19th international conference on e-health networking, applications and services (Healthcom), 2017;1–4. IEEE.

[35] He K, Zhang X, Ren S, Sun J. Deep residual learning for image recognition. In: Proceedings of the IEEE conference on computer vision and pattern recognition, 2016;770–778.

[36] Szegedy C, Ioffe S, Vanhoucke V, Alemi AA. Inception-v4, inception-resnet and the impact of residual connections on learning. In: Thirty-first AAAI conference on artificial intelligence, 2017.

[37] Yu F, Koltun V. Multi-scale context aggregation by dilated convolutions. arXiv preprint arXiv:1511.07122; 2015.

[38] Wang Q, Wu B, Zhu P, Li P, Zuo W, Hu Q. Eca-net: efficient channel attention for deep convolutional neural networks, 2020 IEEE. In: CVF conference on computer vision and pattern recognition (CVPR). IEEE, 2020.

[39] Chen L-C, Papandreou G, Kokkinos I, Murphy K, Yuille AL (2017). DeepLab: Semantic image segmentation with deep convolutional nets, atrous convolution, and fully connected CRFs. IEEE Trans Pattern Anal Mach Intell.

[40] Chaurasia A, Culurciello E. Linknet: Exploiting encoder representations for efficient semantic segmentation. In: 2017 IEEE visual communications and image processing (VCIP), 2017;1–4. IEEE.

[41] Peng C, Zhang X, Yu G, Luo G, Sun J. Large kernel matters–improve semantic segmentation by global convolutional network. In: Proceedings of the IEEE conference on computer vision and pattern recognition, 2017;4353–4361.

[42] Xiang T, Zhang C, Liu D, Song Y, Huang H, Cai W. Bio-net: Learning recurrent bi-directional connections for encoder-decoder architecture. In: International conference on medical image computing and computer-assisted intervention, 2020;74–84. Springer.

[43] Paszke A, Gross S, Chintala S, Chanan G, Yang E, DeVito Z, Lin Z, Desmaison A, Antiga L, Lerer A. Automatic differentiation in pytorch, 2017.

[44] Gu Z, Cheng J, Fu H, Zhou K, Hao H, Zhao Y, Zhang T, Gao S, Liu J (2019). Ce-net: Context encoder network for 2d medical image segmentation. IEEE Trans Med Imaging.

[45] Wan T, Zhao L, Feng H, Li D, Tong C, Qin Z (2020). Robust nuclei segmentation in histopathology using ASPPU-net and boundary refinement. Neurocomputing.

[46] Liu X, Guo Z, Cao J, Tang J (2021). Mdc-net: a new convolutional neural network for nucleus segmentation in histopathology images with distance maps and contour information. Comput Biol Med.

